# Cryotherapy-Enhanced Chemowrap Treatment of Squamous Cell Carcinoma: A Case Series

**DOI:** 10.7759/cureus.15148

**Published:** 2021-05-21

**Authors:** Jason T Bard, Heather A Kornmehl, Lawrence K Chang

**Affiliations:** 1 Department of Dermatology, Eastern Virginia Medical School, Norfolk, USA; 2 Mohs Surgery, Pariser Dermatology Specialists, Norfolk, USA

**Keywords:** 5-fluorouracil, squamous cell carcinoma (scc), cryotherapy, chemowrap, chemotherapeutic wrap, skin cancer, unna boot, actinic keratosis, mohs surgery

## Abstract

An estimated 20% of all malignant cutaneous neoplasms are diagnosed as squamous cell carcinoma (SCC). Chemotherapeutic wraps, or chemowraps, consist of application of topical 5-fluorouracil (5-FU) 5% cream along with occlusive zinc oxide and a compressive bandage (e.g., Unna boot). This treatment modality is often used as a less invasive option compared to surgery, especially in the presence of numerous SCCs. Cryotherapy, the use of liquid nitrogen gas, can be utilized to obliterate pre-malignant and malignant skin lesions. In this report, we present four cases in which females between the ages of 65 and 80 with multiple lower extremity SCCs were treated with cryotherapy prior to each chemowrap application, resulting in favorable clinical tumor improvement. Our observations indicate that cryotherapy may enhance the effectiveness of chemowrap treatment when used before each application. To our knowledge, the use of cryotherapy to synergistically enhance the efficacy of chemowraps has not yet been reported. We hypothesize that cryotherapy induces edema and first strips the outer, hyperkeratotic layers of skin, which facilitates deeper penetration of the 5-FU cream from chemowraps. Chemowraps may also relieve the pain associated with cryotherapy. Therefore, dual cryotherapy and chemowrap treatment may be considered to maximize skin penetration, thus minimizing the extent of surgical intervention in patients with a significant number of SCC lesions.

## Introduction

Approximately one million patients have cutaneous squamous cell carcinoma (SCC) each year, with less than 10% being on the lower extremity [1,2]. Unfortunately, when patients have an abundance of SCC tumors on the lower extremity, surgical options become more limited. Diffuse SCCs on the extremities are typically ill-defined and require extensive surgery [3]. As such, alternative treatment modalities may need to be pursued. One such modality typically used is chemowraps, in which topical 5-fluorouracil (5-FU) 5% cream (e.g., Efudex) along with zinc oxide is applied under occlusion and bandage-wrapped around the patient’s lower extremity for approximately five to seven days each week. The SCC-impacted lesions are typically cleansed and replaced every week for at least four weeks and up to 12 weeks [4,5]. The mechanism behind chemowraps is thought to be related to induction of cellular apoptosis. 5-FU is a cytotoxic agent which binds to thymidylate synthetase to block uracil incorporation into nuclear RNA, thus obliterating cancerous cells [6].

Cryotherapy is also an accepted modality for treating superficial malignancies. Cryotherapy induces edema through osmotic cell injury by forming ice crystals that induce cell lysis, while inflammation is stimulated through tissue ischemia [7]. It has been suggested that combined therapy of liquid nitrogen cryotherapy and 5-FU may produce desired cosmetic outcomes and shorter treatment duration with minimal side effects in basal cell carcinoma (BCC) [8]. To our knowledge, use of cryotherapy to enhance the efficacy of chemotherapeutic wraps in SCC has not yet been reported. In this series, we report four cases in which diffuse lower extremity SCCs were treated with cryotherapy prior to applying chemowraps, resulting in favorable clinical tumor improvement. Cryotherapy facilitates the removal of hyperkeratotic areas, allowing for the penetration of 5-FU in thinner lesions. Therefore, it can augment the efficacy of the 5-FU chemowrap in SCC and diffuse actinic keratosis, particularly in the lower extremities more prone to hyperkeratosis.

## Case presentation

Patient 1 is a 65-year-old female status post Mohs micrographic surgery for SCC of her right lateral heel. The patient also has multiple actinic keratoses on bilateral lower extremities. For the SCC on the right heel, she was continuing treatment with 5-FU and an Unna boot (special gauze impregnated with zinc oxide). Before the 5-FU and Unna boot were reapplied, right leg lesions were treated with liquid nitrogen cryotherapy to generate edema, and thus enhance medication penetration. The patient then returned weekly for seven additional applications, which were well tolerated by the patient. After approximately two months with this intervention, the SCC lesions of the right heel resolved.

Patient 2 is an 80-year-old female who presented with multiple chronic SCCs on bilateral lower legs with an unknown onset. She was started on weekly treatment of cryotherapy with subsequent application of 5-FU and an Unna boot for eight consecutive weeks on the bilateral lower legs. One month later, the patient had multiple resolved keratotic papules on the lower legs. No signs of pustular drainage, redness, or pain were present. There was no further need for additional treatment.

Patient 3 is a 75-year-old female who required an Unna boot application on the bilateral lower legs for multiple areas of actinic keratosis and SCC. Before the Unna boot application, cryotherapy was also performed on the bilateral lower legs to stimulate edema and thereby enhance penetration of 5-FU. Two months later, the bilateral lower extremity keratoses clinically resolved with minimal scarring. No signs of pustular drainage, redness, or pain were present. The patient was then treated with red light Ameluz photodynamic therapy for the next three weeks. A remaining SCC tumor (1.4 × 1.4 cm) of the left leg was removed by Mohs surgery without complications.

**Figure 1:**
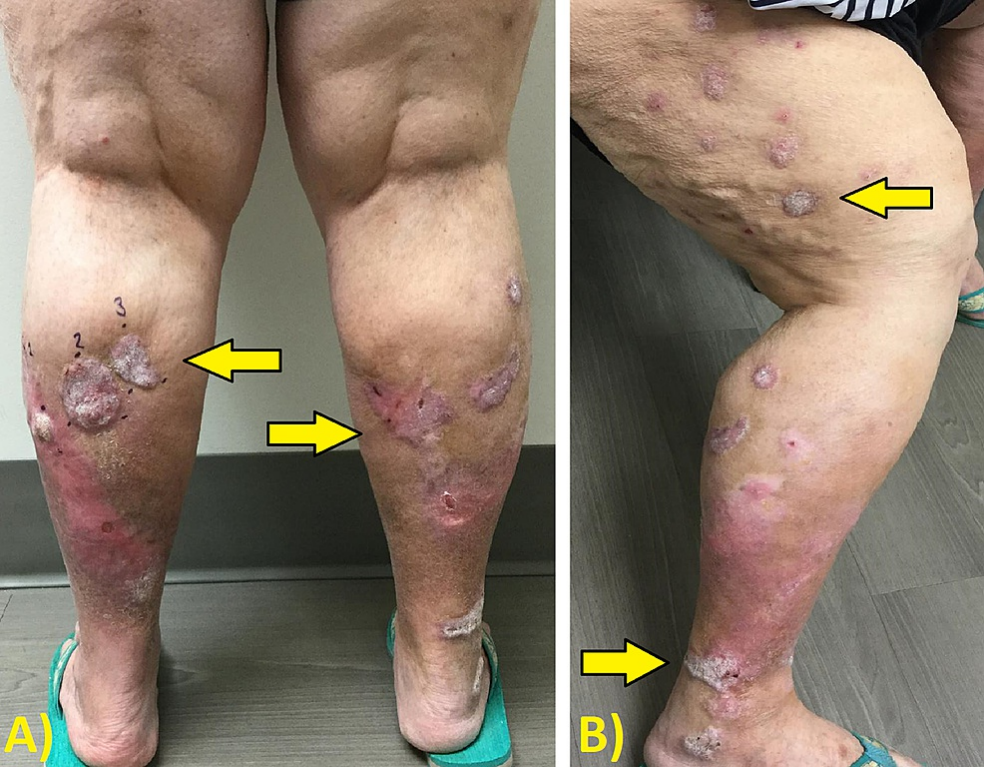
Patient 4 before cryotherapy and concurrent chemowrap treatment. (A) Posterior view and (B) lateral view. Arrows highlight the sites of interest.

**Figure 2:**
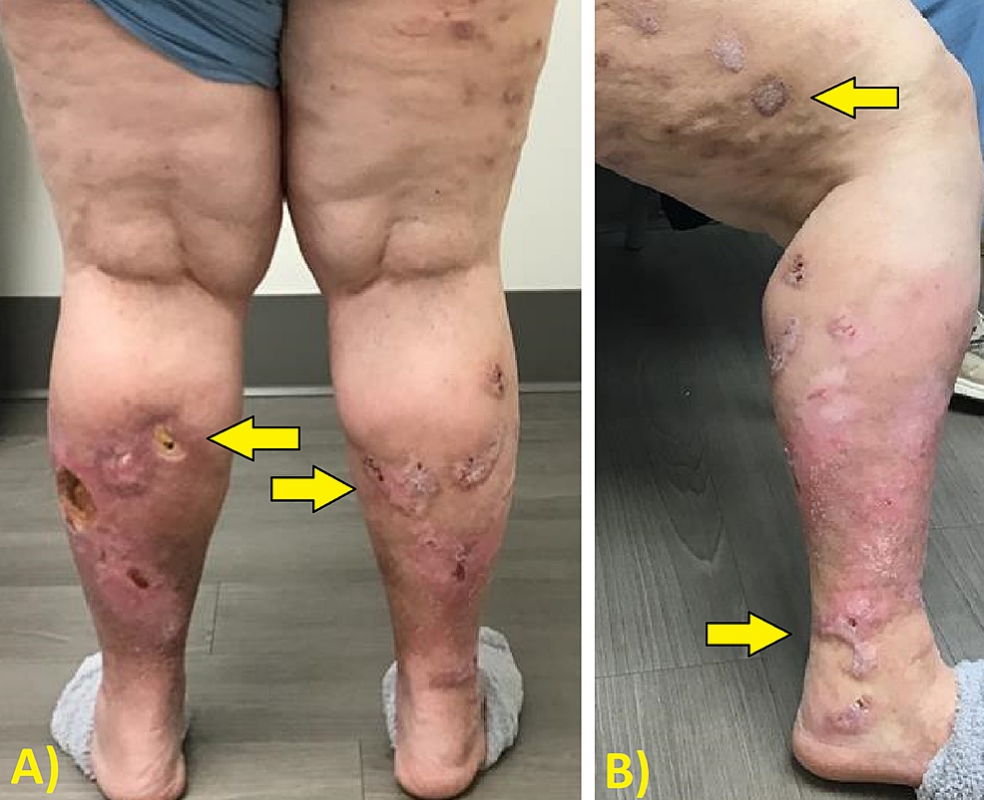
Patient 4 status post eight weeks of cryotherapy with concurrent chemowrap treatment. (A) Posterior view and (B) lateral view. Arrows highlight the sites of interest.

Patient 4 is a 67-year-old female who had a history of SCC about three years prior that was removed by Mohs surgery. She presented with multiple SCCs on the bilateral lower legs for a surgical consultation, as shown in Figure 1. Initiating treatment with cryotherapy and chemowraps was preferred by the patient as opposed to surgery. Weekly treatment of cryotherapy with subsequent application of 5-FU and an Unna boot for eight consecutive weeks on the bilateral lower legs was performed. Notable improvement of the lesions on the lower extremities was seen bilaterally, with only ulceration remaining, as shown in Figure 2. Biopsies of multiple sites confirmed no remaining SCCs. She was then referred to plastic surgery for skin grafting of the ulcers.

## Discussion

SCC accounts for about 20% of all skin cancer diagnoses in the United States. There has been up to a 200% rise in incidence in the past three decades with consistent annual increases [1]. SCC can become highly malignant, with painful and pruritic wounds resistant to healing [9]. Up to approximately 12,600 individuals with cutaneous SCCs develop nodal metastases in a year, resulting in an estimated 9,000 deaths annually [1]. Factors associated with metastasis include tumor diameter, depth, perineural involvement, and location of malignancy [1,10].

Cryotherapy is an effective, non-invasive method which utilizes liquid nitrogen to induce tissue damage through subzero temperatures. Due to cryotherapy’s effectiveness in removing hyperkeratotic lesions, it has the potential to optimize 5-FU absorption into the remaining deeper layers of the skin [4]. Chemowraps typically use topical 5-FU 5% cream as the chemical treatment applied to the SCC. Chemotherapeutic wraps (“chemowraps”) have been demonstrated to prevent SCC metastasis, especially in immunosuppressed patients [4,6]. Another benefit of using cryotherapy concurrently with chemowraps is that cryotherapy can result in ulceration and pain, whereas chemowraps are relatively non-painful and may even alleviate these side effects [6]. We theorize that cryotherapy’s effectiveness in removing hyperkeratotic lesions optimizes 5-FU absorption into the dermis.

After observing the clinical improvement in these four cases, we deduce that it may be beneficial to supplement 5-FU chemotherapeutic wraps with cryotherapy for the treatment of lower extremity cutaneous SCCs. This is especially important when there are a variety of hyperkeratotic lower extremity lesions. Without the use of cryotherapy, 5-FU treatment alone may not be effective in completely resolving lower leg lesions. Further investigation and clinical implementation of this method should be considered in detail.

## Conclusions

The presence of multiple SCCs on the lower extremity at one time can cause discomfort and morbidity among affected patients. Because surgical options may be suboptimal in these clinical scenarios, a topical treatment such as 5-FU is often prescribed to obliterate cancerous cells. With this in mind, chemowraps are preferred in more severe cases involving several SCCs. We have found that the use of liquid nitrogen cryotherapy before routine application of chemowraps appears to improve the rate of healing and appearance of the skin post-treatment. As such, clinicians can consider incorporating cryotherapy into their chemowrap treatment regimen for SCC.
